# Process and components of disaster risk communication in health systems: A thematic analysis

**DOI:** 10.4102/jamba.v14i1.1367

**Published:** 2022-12-08

**Authors:** Arezoo Dehghani, Zohreh Ghomian, Sakineh Rakhshanderou, Hamidreza Khankeh, Amir Kavousi

**Affiliations:** 1 Department of Health in Disasters and Emergencies, School of Public Health and Safety, Shahid Beheshti University of Medical Sciences, Tehran, Islamic Republic of Iran; 2 Department of Health Education and Health Promotion, School of Public Health and Safety, Shahid Beheshti University of Medical Sciences, Tehran, Islamic Republic of Iran; 3 Department of Health in Emergency and Disaster, University of Social Welfare and Rehabilitation Sciences, Tehran, Islamic Republic of Iran; 4 Department of Clinical Science and Education, Karolinska Institute, Stockholm, Sweden; 5 Department of Epidemiology, School of Public Health and Safety, Shahid Beheshti University of Medical Sciences, Tehran, Islamic Republic of Iran

**Keywords:** risk communication, emergency communication, disaster communication, health system, community engagement, disaster management

## Abstract

Risk communication (RC) is one of the necessary functions in disaster management. Establishing communication processes such as planning, transparency of policies and guidelines, RC expert training, providing communication infrastructure and evaluation in the shortest period reduces confusion and management inconsistency. One of the existing challenges is not knowing the exact dimensions of risk communication and its components in disasters. The aim was to identify the components of disaster risk communication in the health system. This research was conducted by systematic review and searching of the databases of PubMed, Scopus, Web of Science, ProQuest, Google Scholar and ScienceOpen 2000–2021 to identify the components of disaster risk communication in the health system. Thematic content analysis was used for data analysis. A Preferred Reporting Items for Systematic Reviews and Meta-Analyses (PRISMA) 2020 chart was used for systematic search, and a modified Strengthening the Reporting of Observational Studies in Epidemiology (STROBE) was used for quality determination. Out of 12 342 articles extracted, 25 studies were included for analysis. The components of disaster risk communication were analysed in 6 categories and 19 subcategories. These categories include communication (communication processes, communication features and infrastructure), information (content production, content characteristics and publishing), risk communication management (risk perception assessment, planning, coordination and logistics), monitoring and control (monitoring and evaluation, accreditation, documentation), education and training (public and organisational) and ethics and values (culture and social beliefs, ethics and trust). According to this research, the establishment of communication infrastructure and advanced equipment such as various structured formats for communication and artificial intelligence; online and offline communication support systems; and timely and accurate notice can help achieve goals such as coordination and organisation in the health system and increase social participation.

**Contribution:**

This study has clarified and explained all the main components and measures of risk communication that can be used for planning scientifically.

## Introduction

In the year 2020, a minimum of 389 natural disasters were reported in the Emergency Events Database (EM-DAT) which caused the deaths of 15 080 people, affecting 98.4 million people and causing $171.3 billion in economic loss (Centre for Research on the Epidemiology of Disasters [CRED] 2020). Damages caused by disasters in low-income countries and gross domestic product (GDP) per capita have always been higher than $2 282.55 (2020) (Kadkhodamanesh et al. 2020).

The effects of disasters in many cases lead to complex and catastrophic situations. Apart from the magnitude and severity of disasters, some of their effects are because of the type of organisation, management and performance of communities (Kirschenbaum 2019). Effective communication, accurate information and analysis of potential risks can play an important role in prevention, risk reduction and right decision-making (Shrestha et al. 2021). Communication processes and tools, information and communication technologies, as well as mass media, early warning systems and public education about emergencies are among the key and important measures in managing these events and reducing losses (Tinker et al. 2011) As a lesson learned from many disasters, risk communication has also been identified as a key factor in planning and responding to emergencies, and coordination and communication in the successful management of disasters has also been emphasised (Ow Yong et al. 2020).

This situation requires measures such as rescue, providing health services in prehospital and hospital, communicable and noncommunicable diseases, environmental health, family and population health, nutrition, psychosocial support, health education, laboratory services and drug supply. It is imperative for health managers, as the first decision-makers and respondents, to communicate, answer questions from senior officials, the community and journalists and interact effectively with all levels of administration and society. It should be done in the shortest time (Khangah et al. 2017). These measures are defined in the process of risk communication, which is one of the efficient components in all stages of the disaster risk management cycle from preparedness to response and rehabilitation (Khangah et al. 2017).

The process of risk communication in countries is different, and it is influenced by many factors and includes various components. In the preliminary search, systematic review studies are the subject of this research (Sato et al. 2020). One of the existing problems is the lack of transparency of risk communication components, and the clarity of risk communication components will help to plan accurately, comprehensively and adaptively. This study aims to provide a comprehensive overview to identify the components of disaster risk communication in the health system for principled and effective management of emergencies, reducing parallel work, community engagement and rumors management.

## Methodology

This study was conducted by systematic review with the Prospero code CRD42021268686. Articles were included by searching in international databases such as PubMed, ProQuest, Scopus, Web of Science, Google Scholar and ScienceOpen.

## Search strategy

**FIGURE 1:**
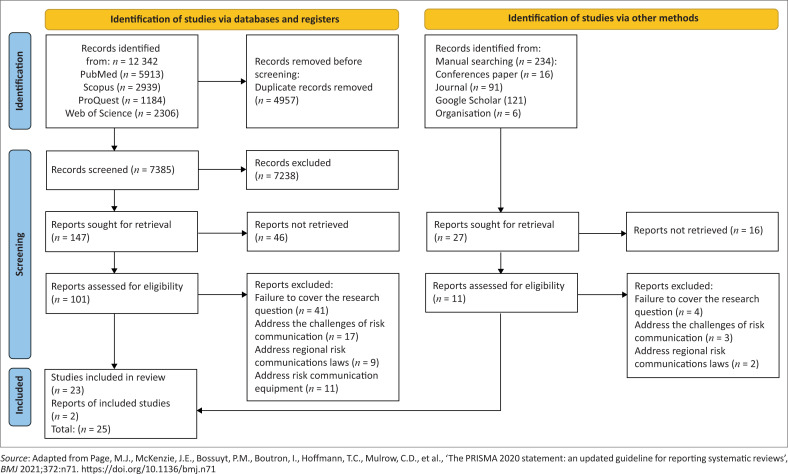
Preferred Reporting Items for Systematic Reviews and Meta-Analyses (PRISMA) chart 2020 systematic study.

A Preferred Reporting Items for Systematic Reviews and Meta-Analyses (PRISMA) chart 2020 (Figure 1) was used for systematic research (Page et al. 2021). According to the title and subject of research, the relevant keywords were determined and finalised by the research team based on the initial review of the texts, use of MeSH by the NCBI database, the use of keywords of related articles and the opinions of experts.

This was done by using the keywords ‘risk communication’, ‘disaster’ and ‘health system’. Also, for communication synonyms, words such as ‘communicative’, ‘communicating’, ‘communicator’, ‘communicational’ and ‘communicatively’ were used. For the word ‘disaster’, synonyms such as ‘emergency’, ‘crisis’ and ‘pandemic’ were also used. For the term ‘health systems’, synonyms such as ‘healthcare delivery’, ‘healthcare’, ‘healthcare system’, ‘health service’, ‘health sector’, ‘public health system’, ‘health delivery system’, ‘health field’ and ‘public health’ were used.

To design the search strategy, ‘OR’ operators and ‘AND’ operators were used. In the end, an exclusive research strategy was designed for each main database. Thereafter, by testing the relevance index of articles on the search page in the database (Table 1) and separate steps, the filters of title, abstract and their combination were used.

**TABLE 1:** Search strategy to identify the disaster risk communication components in world health systems.

Data base	Syntax
PubMed	(((Risk) AND (communication)) OR (“Risk communication”[tiab]) OR (“risk communicating”) OR (“risk communicator”) OR (“risk communications”[tiab]) OR (“risk communicational”) OR (“risk communicatively”) OR (“risk communicativeness”) OR (“risk communicators”) OR (“emergency communication”) OR (“emergencies communication”[tiab]) OR (“disaster communication”) OR (“disasters communication”)) AND ((Disaster[tiab]) OR (disasters) OR (emergency) OR (emergencies) OR (crisis) OR (pandemic) OR (pandemics)) AND ((“health system”[tiab]) OR (“health systems”) OR (“health care system”[tiab]) OR (“health care systems”) OR (“health care”) OR (“health care delivery”) OR (“public health”) OR (“health field”) OR (“health delivery system”) OR (“public health system”) OR (“health sector”) OR (“health care sector”) OR (“health service”)) AND (2000:2021[pdat])
Scopus	(TITLE-ABS(( risk ) AND ( communication )) OR TITLE ( “Risk communication” ) OR TITLE ( “risk communicating” ) OR TITLE-ABS(( risk ) AND ( communicating )) OR TITLE-ABS( “risk communicator” ) OR TITLE( “risk communications” ) OR TITLE-ABS(( risk ) AND ( communications )) OR TITLE-ABS( “risk communicational”) OR TITLE-ABS(( risk) AND ( communicational)) OR ALL ( “risk communicatively” ) OR TITLE-ABS( “risk communicativeness” ) OR ALL( “risk communicators” ) OR TITLE-ABS(( risk) AND ( communicators )) OR TITLE( “emergency communication” ) OR TITLE-ABS ( “emergencies communication”) OR TITLE ( “disaster communication” ) OR TITLE ( “disasters communication” )) AND ( TITLE ( disaster) OR TITLE ( disasters ) OR TITLE-ABS( emergency ) OR TITLE-ABS( emergencies ) OR TITLE-ABS( crisis ) OR TITLE-ABS( pandemic ) OR TITLE-ABS( pandemics )) AND ( TITLE ( “health system” ) OR TITLE-ABS( “health systems”) OR TITLE( “health care system”) OR TITLE-ABS(( “health care” ) AND ( system)) OR TITLE-ABS( “health care systems”) OR TITLE-ABS(( “health care” ) AND ( systems )) OR TITLE-ABS( “health care” ) OR TITLE ( “health care delivery” ) OR ALL ( “public health” ) OR ALL ( “health field”) OR ALL ( “health delivery system”) OR TITLE-ABS( “health delivery” ) OR TITLE-ABS( “public health system” ) OR TITLE-ABS( “health sector” ) OR TITLE-ABS( “health care sector” ) OR TITLE-ABS(( “health care” ) AND ( sector )) OR TITLE ( “health service” )) AND ( PUBYEAR > 2000 )
Proquest	(ALL((Risk) AND (communication)) OR AB,TI(“Risk communication”) OR AB,TI(“risk communicating”) OR ALL((risk) AND (communicating)) OR ALL(“risk communicator”) OR AB,TI(“risk communications”) OR ALL((Risk) AND (communications)) OR ALL(“risk communicational”) OR ALL((Risk) AND (communicational)) OR ALL(“risk communicatively”) OR ALL(“risk communicativeness”) OR ALL(“risk communicators”) OR ALL((Risk) AND (communicators)) OR AB.TI (“emergency communication”) OR AB,TI (“emergencies communication”) OR AB,TI(“disaster communication”) OR AB,TI(“disasters communication”)) AND (ALL (Disaster) OR ALL (disasters) OR ALL(emergency) OR ALL(emergencies) OR ALL(crisis) OR ALL(pandemic) OR ALL(pandemics)) AND (AB,TI (“health system”) OR AB.TI (“health systems”) OR AB.TI (“health care system”) OR ALL((“health care”) AND (system)) OR ALL(“health care systems”) OR ALL((“health care”) AND (systems)) OR ALL(“health care”) OR ALL (“health care delivery”) OR ALL(“public health”) OR ALL(“health field”) OR ALL(“health delivery system”) OR ALL(“health delivery”) OR ALL(“public health system”) OR ALL(“health sector”) OR ALL(“health care sector”) OR ALL((“health care”) AND (sector))OR ALL(“health service”)) AND YR(20000101-20211230)
WoS/ WoK	(TS=((Risk) AND (communication)) OR TI=(“Risk communication”) OR TI=(“risk communicating”) OR TI=((risk) AND (communicating)) OR TI=(“risk communicator”) OR TI=(“risk communications”) OR TS=((Risk) AND (communications)) OR TS=(“risk communicational”) OR TS=((Risk) AND (communicational)) OR TS=(“risk communicatively”) OR TS=(“risk communicativeness”) OR TS=(“risk communicators”) OR TS=((Risk) AND (communicators)) OR TI=(“emergency communication”) OR TI=(“emergencies communication”) OR TI=(“disaster communication”) OR TI=(“disasters communication”)) AND (TS= (Disaster) OR TS= (disasters) OR TS=(emergency) OR TS=(emergencies) OR TS=(crisis) OR TS=(pandemic) OR TS=(pandemics)) AND (TS (“health system”) OR TS (“health systems”) OR TS (“health care system”) OR TS=((“health care”) AND (system)) OR TS=(“health care systems”) OR TS=((“health care”) AND (systems)) OR TS=(“health care”) OR TS= (“health care delivery”) OR TS=(“public health”) OR TS=(“health field”) OR TS=(“health delivery system”) OR TS=(“health delivery”) OR TS=(“public health system”) OR TS=(“health sector”) OR TS=(“health care sector”) OR TS=((“health care”) AND (sector))OR TS=(“health service”)) AND PY=(2000-2021)

WoS, Web of Science; WoK, Web of Knowledge.

## Article selection process

### Article inclusion criteria

Studies that are relevant to disaster risk communications in the health system from 2000 to 2021, available in full text and have high quality in method and design were included in this systematic review.

### Article exclusion criteria

Articles dealing with various other aspects of disaster risk communication, such as communication between medical teams, communication between patients and medical staff, the challenges of risk communication and articles whose full text was not available, were excluded from the study.

Duplicate studies and then the titles and abstracts of each study were deleted in terms of relevance and unrelated studies. In the full-text selection stage, the screened studies were retrieved, and the studies whose full text was not available were deleted. Then, the process of selecting studies was done by studying the full text of the article based on the inclusion and exclusion criteria of this study. At this stage, two members of the research team, after reading the full text of each study independently, divided the studies into three categories: imported, exported and borderline. After determining the cases of agreement, the decision for disagreement was made through the decision of the third person and reaching a consensus between the research team, and finally, the selected articles included in this study.

### Collecting data stages

After finalising the articles submitted to the research databases, the extracted data were entered into a Microsoft Excel file (Microsoft Corporation, Redmond, Washington, United States) which included the name of the journal, article title, year of publication, author name, country name, research method and study results for evaluation and analysis. The main features of the studies and their results are shown in Table 3.

**TABLE 2:** Construction of the researcher quality assessment checklist.

Area	Question	Answer		
		Yes (1)	No (0)	Unknown
Title	Does the title of the article match the research question and method?	-	-	-
Introduction	Are the objectives of the study clear in the introduction?	-	-	-
	Is there an introduction to the existing gaps and the importance of the issue?	-	-	-
Methodology	Is the exact methodology specified?	-	-	-
	Is the study setting described?	-	-	-
	How is the data collected transparent?	-	-	-
	Is the data collection tool described?	-	-	-
	Is the process and method of data analysis described?	-	-	-
Findings	Are the findings in line with the objectives of the study?	-	-	-
	Are the findings fully described?	-	-	-
	Are the findings generalisable and practical?	-	-	-
Discussion and conclusion	Key results discussed?	-	-	-
	Are the strengths and weaknesses well described?	-	-	-

**TABLE 3:** Characteristics of the articles included in the systematic review.

Author’s name	Year	Article title	Type	Design	Finding focus of articles
Vincent Covello	2003	Best practices in public health risk and crisis communication (Covello 2003)	Mixed methods	Literature review, content analysis	Stakeholder participation; Use of all communication channels; Observance of ethical standards; Feedback on people’s opinions and questions; Providing honest and transparent information; Completing the information vacuum; Non-confidentiality; Synchronising internal and external communication; Using graphic images; Skills training; Basic, intermediate and advanced communication
Terrence A. Maxwell	2003	The public need to know: emergencies, government organizations, and public information policies (Maxwell 2003)	Mixed methods	Case study	Study of public response in emergencies; Mutual trust between government and people; Empathy with people; Determining the level of authority and decision-making; Collection and processing and dissemination of information; Management of government emergency information, network features, understanding how issues are framed and information flows in units; Information flow responsibility; Information gateway responsibility issues
Mike Granatt	2004	On trust: Using public information and warning partnerships to support the community response to an emergency (Granatt 2006)	Qualitative	Document analysis	Having active communication before, during and after the accident; Providing evidence behind decisions; Ensuring that information is provided by credible sources; Integrity of information; Freedom of the media; Operational planning; Use of simple language; Exchange of lessons learned; Planning for media centres; Media accreditation schemes
John Hobbs, Anne Kittler, Susannah Fox, Bates	2004	Communicating health information to an alarmed public facing a threat such as a bioterrorist -attack (Hobbs et al. 2004)	Mixed methods	Literature review, content analysis	Effective communication between scientists and service providers and managers; Using traditional social media; Using the main website of responsible organisations; Determining alternative information sources; Increasing access to quality information; Developing transparent organisational communication protocols
Branden B. Johnson, Caron Chess	2006	From the inside out: Environmental agency views about communications with the public (Johnson & Chess 2006)	Qualitative	Content analysis	Develop a public communication plan; Implement a communication plan with public culture; Commitment of managers to implement the plan; Avoid politicisation in risk communication; Existence of knowledge and skills in managers and risk communication experts; Develop appropriate regulations for establishing risk communication; Establish regular communication with citizens
Marsha L. Vanderford, Teresa Nastoff, Jana L. Telfer and Sandra E. Bonzo	2007	Emergency communication challenges in response to Hurricane Katrina: Lessons from the Centers for Disease Control and Prevention (CDC) (Vanderford et al. 2007)	Mixed methods	Strategy analysis, content analysis	Up-to-date educational messages; Up-to-date information sites; Access to Internet, electricity and telephone infrastructure; Use of alternative communication channels; Localisation of communication measures; Analysis of audiences; Utilisation of health and educational communication capacity in local organisations
Ricardo J. Wray, Steven M. Becker, Neil Henderson, Deborah Glik	2009	Communicating with the public about emerging health threats: Lessons from the Pre-Event Message Development Project (Wray et al. 2008)	Qualitative	Formative research, focus group	Informing about public priority actions; Managing anxiety, fear, shock, anger and disbelief; Building trust in the government; Providing accurate and timely information; Addressing consistent information needs; Responding to the media; Coverage and emergency information
Bev J. Holmes, Natalie Henrich, Sara Hancock	2009	Communicating with the public during health crises: Experts’ experiences and opinions (Holmes et al. 2009)	Qualitative	Content analysis	Empowering people; Regularly updating the media; Changing attitudes and behaviours as a result of following general guidelines; Transmitting information about high-risk groups without stigmatising; Paying attention to ethical challenges in communication
Elaine Vaughan And Timothy Tinker	2009	Effective health risk communication about pandemic influenza for vulnerable populations (Vaughan & Tinker 2009)	Qualitative	Document analysis	Strategic planning for risk communication; Rumour management; Attention to psychological, social, cultural, health and socio-economic dimensions; Establishing step-by-step communication; Simple, feasible communication solutions; Interaction with social organisations, researchers and other individuals and agencies; Communication planning
Lenette Golding	2010	Training for public information officers in communication to reduce health disparities: A needs assessment (Golding & Rubin 2011)	Quantitative	Needs assessment survey	Launching a campaign, preparing a newsletter, brochure; Coordination within and between organisations; Developing working relationships with the media, civil and social organisations and the people; Monitoring the establishment of risk communication; Conducting risk communication manoeuvres; Allocating and providing resources; Using information technology
Seyed Hesam Seyedin and Hamid R. Jamali	2011	Health information and communication system for emergency management in a developing country, Iran (Seyedin & Jamali 2011)	Mixed methods	Qualitative analysis, literature review	Effective communication between employees and managers; Knowledge of key people; Effective collection of data and information; Development of standards and communication protocols; Use of information technology; Existence of advanced database; Existence of intelligent system for data collection; Comprehensive analysis of information for decision-making
Ezequiel M. Galarce, K. Viswanath	2012	Crisis communication: An inequalities perspective on the 2010 Boston water crisis (Galarce & Viswanath 2012)	Quantitative	Online survey	Determining reliable sources of information; Evaluating information sources by the recipients of the message; Using social networks; Preparing risk communication scenarios and training and practice; Reconstructing one’s thoughts and feelings
Felicia Mebane, Sarah Temin and Claudia F. Parvanta	2014	Communicating anthrax in 2001: A comparison of CDC information and print media accounts (Mebane, Temin & Parvanta 2003)	Qualitative	Content analysis	Dissemination of information– accuracy of objective information–conceptualisation of disaster communication–preparation of news reports for the public, television and Internet news sources–analysis of emergency communication performance–better cooperation between departments, sharing of trusted information and communication between different administrative levels
Petra Dickmann, A. McClelland, Gaya M. Gamhewage, Patricia Portela de Souza, Franklin Apfel	2015	Making sense of communication interventions in public health emergencies: An evaluation framework for risk communication (Dickmann et al. 2015)	Mixed methods	Qualitative analysis and literature review	Understanding the risk of officials and people; Institutionalising the risk communication process; Establishing coordination between risk communication components; Evaluating the process of collecting, reviewing and disseminating information; Communication and local coordination, national and international regions; Selecting reliable platforms and channels for disseminating information
Elena Savoia, Leesa Lin, Gaya M. Gamhewage	2017	A conceptual framework for the evaluation of emergency risk communications (Savoia et al. 2017)	Mixed methods	Literature review, content analysis	How and when to present policy change information with respect to mitigation strategies and social consequences, economic implications for the dissemination of information on public health threats; risk assessment findings into information and messages; system ability to use existing programmes; knowledge transfer to Beneficiary organisations
Matthew L. Spialek, J. Brian Houston	2018	The development and initial validation of the citizen disaster communication assessment (Spialek & Houston 2018)	Mixed methods	Systematic review, comprehensive survey	Using social media for documentation and education; Disaster communication ecology; Citizens’ communication; Encouraging people to correct rumours; Access warning messages; Expressing individual and organisational experiences; Considering horizontal communication between residents at the micro level; Formal pre-event communication strategies; Attention to mental health
Matthew W. Seeger, Laura E. Pechta, Simani M. Price, Keri M. Lubell	2018	A conceptual model for evaluating emergency risk communication in public health (Savoia et al. 2017)	Mixed methods	Literature review, content analysis	Provide emergency risk communication messages from various channels, including social media and websites, websites; direct questions; increase message coordination to increase stability; information coherence; implementation of guidelines; information trust
Pradeep Sopory, Ashleigh M. Day, Julie M. Novak	2019	Communicating uncertainty during public health emergency events: A systematic review (Sopory et al. 2019)	Mixed methods	Multiplemethods, evidence synthesis	Actively generate information to reduce uncertainty; Complete information gaps; Pay attention to personal networks; Respond to alerts; Disseminate accurate information through networks of physicians, nurses, community leaders; Regular information about event progress through mass media
Zeinab Bagheri, Tahere Dehdari, Masoud Lotfizadeh	2020	Psychometrics of emergency risk communication checklist in public health sector (Bagheri, Dehdari & Lotfizadeh 2020)	Quantitative	Factor analysis	Identification of media outlets to the public, stakeholders and partner organisations; Development of an action plan for information and communication with the media; Existence of specific paths and responsibilities for information group staff; Coordination with responsive teams; Accurate assessment of information needs of media, partner and public organisations
Natalie Bernard, Abdul Basit, Ernesta Sofija, Jessica Lee, Shanon Rutherford	2021	Analysis of crisis communication by the Prime Minister of Australia during the COVID-19 pandemic (Bernard et al. 2021)	Qualitative	Content analysis	Empathic behaviour of officials with the people; Clarification of the current situation; Compilation of key messages of public education before the crisis; Report of actions; Training of leaders and risk communication specialists
Hui Zhang, Yingxiang Li, Chris Dolan, Zhijun Song	2021	Observations from Wuhan: An adaptive risk and crisis communication system for a health emergency (Zhang et al. 2021)	Qualitative	Ethnography	Holding press conferences, online telephones for answering; providing specialised information platform; communication support system; using artificial intelligence software; using experts in press conferences; online and offline communication support systems; public opinion management; using adaptive information system
Dionne Mitcham, Morgan Taylor, Curtis Harris	2021	Utilizing Social Media for Information Dispersal during Local Disasters: The Communication Hub Framework for Local Emergency Management (Mitcham et al. 2021)	Mixed methods	Systematic review and qualitative analysis	Use of social media platforms; Maintain a manageable control domain; Use of social media messaging model; Determine communication framework; Facilitate external and internal communication; Select appropriate media channels
Ratchadaporn Papwijitsil, Pigunkaew Sinam, Mathudara Phaiyarom	2021	Factors Related to Health Risk Communication Outcomes Among Migrant Workers in Thailand during COVID-19 (Papwijitsil et al. 2021)	Quantitative	Case study	Paying attention to the demographic information of the audience; Using different information sources (experts; community members; mass and social media); Understanding the common interests in the community; Access to communication infrastructure; Using supportive resources; Paying attention to the health of the community; Creating flexibility in communication processes
Khayriyyah Mohd Hanafiah, Celine Ng, Abdul Matiin Wan	2021	Effective communication at different phases of COVID-19 prevention: Roles, enablers and barriers (Mohd Hanafiah et al. 2021)	Quantitative	Commentary	Nonbombardment of information; Ensuring the acceptance of public health messages; Scientific communication and culture-building; Combating false information; Identifying and managing false information; Filling gaps with scientific language; Filling the gap of risk perception between professionals and nonspecialists
Krithika Venkatraman and Anand Manoharan	2021	Public engagement as the fifth dimension of outbreak communication: Public’s perceptions of health communication during COVID-19 in India (Venkatraman & Manoharan 2021)	Mixed methods	Qualitative analysis	Creating awareness, two-way communication and encouraging people to accept government recommendations; Creating value for the general public; Aligning risk communication with the people; Planning; Program updating; Promising; Creating two-way communication mechanisms

COVID-19, coronavirus disease 2019; CDC, Centers for Disease Control and Prevention.

### Study risk of bias assessment

The STROBE checklist was used to evaluate the quality of articles (White et al. 2015). The Strengthening the Reporting of Observational Studies in Epidemiology (STROBE) tool was modified according to the nature of the study, which includes 13 items and the answers yes, no and unknown (Table 2). Articles that have a score of 0–3 were weak, 4–8 average and 9–13 strong. Two members of the research team independently evaluated the quality of the full text of the extracted articles, and disagreements were determined by the third person.

### Data analysis

In this research, the thematic analysis method was used to analyse the data, which is one of the qualitative research methods and includes the combination and interpretation of different findings with a focus on the study subject.

Two researchers analysed all the components extracted from the articles. Duplicate components were removed. The remaining list of principal components was studied and evaluated several times. Similar components were classed into subcategories according to thematic similarity. In the next stage, during several times of study and revision, similar subcategories were classed and then the main categories were created.

### Ethical considerations

Ethical approval to conduct this study was obtained from the Shahid Behehshti University of Medical Sciences, Research and Ethics Committee (ref. no. IR.SBMU.PHNS. REC.1400.067).

## Results

### Study selection

According to the PRISMA flowchart, out of 12 342 articles found using the database search strategy, 4 957 articles were deleted because of duplication, 7 238 articles were deleted on account of lack of relevance to the research topic and 41 articles were deleted because of lack of access to the full text. The full text of 112 articles was carefully reviewed and entered the critical review stage. Finally, 25 articles that had average and high quality in evaluation were included in the study using the STROBE modified tool. In this study, 48% of the articles were mixed methods, 32% were qualitative and 20% were quantitative. Articles from China, Australia, India, Britain, the United States (US) and Iran were reviewed in this article. Most of the articles are from the US, Australia and Britain. The highest number of articles was related to the years 2015–2021 (44%).

### Thematic analysis

**FIGURE 2:**
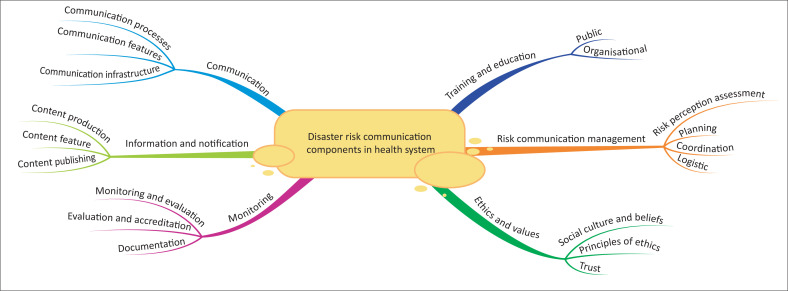
Disaster risk communication components in the health system, extracted from thematic analysis.

Based on the studies included in this systematic review, the elements of disaster risk communication in the health system were divided into 6 main categories and 19 subcategories, which include communications (subcategories: communication processes, communication features and communication infrastructure), information (subcategories: content production, content characteristics and content publishing), risk communication management (subcategories: risk perception assessment, planning, coordination and support), monitoring (subcategories: monitoring and evaluation, monitoring and accreditation, documentation), training and education (subcategories: public and organisational) and human ethics and values (subclasses: culture and social beliefs, justice and ethics, trust) (Figure 2).

#### Communication

Based on the findings, processes and infrastructure, as well as their characteristics, are important components of risk communication.

**Communication processes:** Establishing proper communication with the media before the disaster and maintaining it during the disaster management cycle and effective use of their operational abilities, because of the influence, importance and acceptance of the media in society, is one of the communication process dimensions which needs the necessary planning (Dickmann et al. 2015; Hobbs et al. 2004; Mitcham, Taylor & Harris 2021; Savoia, Lin & Gamhewage 2017; Venkatraman & Manoharan 2021; White et al. 2015). As another layer of the communication process, communication should be established with nonprofit organisations, partners and stakeholders, as well as local health liaisons, and must even establish effective communication between experts and managers (Hobbs et al. 2004; Savoia et al. 2017).

**Communication features:** Communication with the public, media and organisations must have certain characteristics to be sustainable. Creating interactive and continuous communication (Dickmann et al. 2015; Venkatraman & Manoharan 2021); flexible and adaptive communication (Mitcham, Taylor & Harris 2021; Wray et al. 2008); active communication based on public perception; and audience-oriented (Papwijitsil et al. 2021; Vanderford et al. 2007), direct and indirect multidimensional communication (Holmes et al. 2009) have led to the institutionalisation of communications (Dickmann et al. 2015) and should always be considered by risk communication experts in organisations.

**Communication infrastructure:** Information and communication infrastructure and the resulting opportunities lead to the establishment of extensive and highly influential communications and increase the effectiveness of disaster risk communication. Access to basic infrastructures such as the Internet, electricity and telephones is one of the important components in establishing the process of risk communication (Mohd Hanafiah, Ng & Wan 2021; Zhang et al. 2021). Considering the definite possibility of vital lines such as telephones, electricity and Internet, determining online and offline communication support systems and using alternative communication routes according to the type of disasters and the level and community and audience should be considered by organisations (Heath et al. 2018; May 2005; Mohd Hanafiah et al. 2021; Zhang et al. 2021).

#### Information and notification

Information is one of the substantial aspects of disaster risk communication. Disaster risk information has both an educational aspect and a piece of information for the general public and stakeholders that will help organisational decision-making and public behaviour changes. During this systematic study, it was clear that content production, content features and how it is published have significant effects on disaster risk communication in the health system.

**Content production:** Based on the nature of disasters, key messages should be prepared beforehand to minimise the distance between the event and the information publishing (Bernard et al. 2021; Granatt 2004; Spialek & Houston 2018). Content production should be tailored to the needs of the media, and people should be completely honest, transparent and understandable to change the behaviour of society and improve its readiness (Covello 2003; Johnson & Chess 2006; eds. Zhang, Li & Chen 2020).

**Content features:** One of the most important ways to attract the audience is to pay attention to the visual appeal of the message. Also, short information should be provided several times to streamline information (Covello 2003; Vanderford et al. 2007; Zhang et al. 2021). Risk communication should not be influenced by politics (May 2005; Vanderford et al. 2007) and avoid making unenforceable promises and providing too much and confusing information (Covello 2003; May 2005).

**Content publishing:** Content publishing, or the distribution of content, is one of the most important steps in the information process that comes after the production of content phase. It is always recommended to use all mass media as well as local, national and social media for the wide dissemination of disaster information (Spialek & Houston 2018; eds. Zhang et al. 2020). Also, using a capable and specific speaker to describe actions, providing information and holding a press conference to publish content was one of the main strategies (Maxwell 2003; Zhang et al. 2021).

#### Risk communication management

Risk communication management is one of the most significant parts of this process, which in addition to assessing risk perception is also responsible for planning, coordination and support.

**Risk perception assessment:** The first component of planning for risk communication is to assess people’s perception of risk and level of concern to predict the emotional response of society to disasters. In other words, the ecology of risk communication and identification of information needs to be considered (Mohd Hanafiah et al. 2021; eds. Smith et al. 2007; Wray et al. 2008).

**Planning:** Communication planning is a multidimensional concept that leads to the development of a strategic risk communication plan (Golding & Rubin 2011; Zhang et al. 2020). Also, all team members must be able to respond appropriately. Standards and protocols of risk communication (Hobbs et al. 2004; Granatt 2004; Seyedin & Jamali 2011), roadmaps, responsibilities and job descriptions should be developed (Maxwell 2003; Zimmerman et al. 2010). Another effective measure to improve the communication process is a risk assessment and updating program (Dickmann et al. 2016).

**Coordination:** Coordination is one of the pillars of the chain of command and is one of the basic principles of risk communication. For the establishment of a proper risk communication process, communication and coordination at the local, national and international levels must be planned and implemented before the crisis occurs (May 2005; Zhang et al. 2020). It is also necessary to provide a platform for cooperation and interaction with stakeholders, social and nonprofit organisations and researchers (Mitcham et al. 2021; Zhang et al. 2021).

**Logistics:** Supporting the process of establishing risk communications includes providing the necessary resources to carry out activities, manpower, programmes and other management items in this area (Papwijitsil et al. 2021). In the current situation, there is a need to use support resources also (Covello 2003; Hobbs et al. 2004). On the other hand, in disasters, there is a possibility of damage to communication infrastructure such as electricity, telephone and the Internet, so providing a support communication system should be considered (Papwijitsil et al. 2021; Vanderford et al. 2009)

#### Monitoring

Monitoring is one of the components of organisational processes to improve responses and includes monitoring and evaluation, accreditation and documentation.

**Monitoring and evaluation:** The process of risk communication needs to be established in all stages of the disaster management cycle; therefore, it is necessary to continue its evaluation during this cycle (Vanderford et al. 2007; Zhang et al. 2020). Moreover, the activity of communication networks in terms of software and hardware should also be evaluated periodically (Gallardo-Paúls 2021). The information process, as the most important components of risk communication, must also be monitored to ensure access to accurate information, at the right time and in the right way (Dickmann et al. 2015). Finally, incorrect information and rumours must be managed and even removed by identifying them (Gallardo-Paúls 2021; Vanderford et al. 2007).

**Accreditation:** It is necessary to monitor the establishment of risk communication, which always has many challenges (Golding & Rubin 2011). In addition, monitoring the implementation of protocols and providing feedback should be considered by managers and experts from the beginning of the risk communication process to improve decision-making and response (Dickmann et al. 2016). Accreditation of risk communication processes in responsible organisations should be done periodically or annually (Granatt 2004).

**Documentation:** Documenting individual and organisational experiences and lessons learned in risk communication, exchanging lessons learned at national and international levels and using lessons learned in future programmes will enhance the risk communication process (Granatt 2004; Spialek & Houston 2018; White et al. 2015). In some studies, it has been suggested that a monitoring team should be present along with the assessment team from the beginning of the risk communication process (Covello 2003; Johnson 2006).

#### Education

For the establishment and effectiveness of the risk communication process, training at the public and organisational levels should be included in the pre-, mid- and even post-disaster stage of programmes. Public education will help by producing and distributing educational content and media at the community level to bring in positive behaviour, improve risk reduction indicators and increase disaster preparedness among families and the general public (Bagheri et al. 2021; Spialek & Houston 2018). In addition to improving the knowledge of managers and experts in the field of risk communication and their components, concepts, strategies and plans and responsibilities (May 2005), other key people in the organisation should also be made familiar with risk communication processes (Ow Yong et al. 2020; Vanderford et al. 2007). In this regard, risk communication manoeuvres can be planned (Gallardo-Paúls 2021; Golding & Rubin 2011).

#### Ethics and values

Risk communication as a community-oriented process must always include human ethics and values such as culture and social beliefs, trust, justice and ethics.

**Social culture and beliefs:** Economic and social conditions must be considered to plan and formulate risk communication protocols to determine the main sources of public information (Covello 2003; Maxwell 2003). Also, the risk communication plan and content production should be adapted to the culture (Holmes et al. 2009; Vanderford et al. 2007). On the other hand, due to the importance of public opinion management, it should be done while giving hope in the society through effective risk communication and rebuilding the thoughts and feelings of the society (Ow Yong et al. 2020; Spialek & Houston 2018; Zhang et al. 2021).

**Principles of ethics:** One of the measures that should be taken up in the precrisis phase is the development of ethical principles of risk communication (Granatt 2004; Johnson & Chess 2006). Stakeholders and the media must also adhere to moral obligations (Covello 2003; Holmes et al. 2009). It is necessary to learn ethical criteria to avoid embarrassing the people and not blaming or holding other organisations accountable, which affects communication processes.

**Trust:** Risk communication activities, information paths and content produced should all lead to mutual trust between the government and the people to pave the way for the flow of risk communication (Dickmann et al. 2015; Hobbs et al. 2004). Efforts should be made to improve public confidence in official sources of information (Venkatraman & Manoharan 2021; Wray et al. 2008), which will facilitate the management of public opinion, social networks and rumours (Mohd Hanafiah et al. 2021).

## Discussion

The main goal of this study was to extract the components of disaster risk communication in the health system. Based on the findings of this study, information, communication, management, monitoring and evaluation, education and ethics and values are the six main components of disaster risk communication in the health system.

Disaster risk communication is a multidimensional and long-term process that plays a principal role in reducing risk and organisational and extra-organisational preparedness. Effective disaster risk communication lead to right decision-making, increase risk understanding, improve community engagement and preparedness. Explaining the factors and elements of disaster risk communication in the health system leads to an appropriate response based on need, without wasting resources and parallel work, and improving rehabilitation processes and, most importantly, health system resilience in all stages of health management in disasters.

In this research, planning and information are the most important things related to disaster risk. These components require an assessment of the existing situation and planning based on the culture, social and economic issues and health literacy community to create a consistent programme. The process of risk communication provides a more appropriate response to disasters, public behaviour change and evidence-based decisions.

A study by Mebane et al. (2003) showed that if an organisation has even the most sophisticated forecasting models and advanced early warning systems, but the information is not transmitted promptly, it will not allow the end user to understand and act correctly, and finally, it will be useless (Mebane et al. 2003).

These findings concur with the current research. On the other hand, to communicate at different levels and with a wide range of audiences, a differently structured format, specialised and comprehensive communication platforms must be used to ensure the availability of the information (Dickmann et al. 2016; Papwijitsil et al. 2021).

Paying attention to the values as well as the ecology of risk communication with an emphasis on cultural, social, economic and educational issues of the community for audience evaluation, contingency and audience-oriented planning, determining communication channels and information needed to promote risk communication processes and building public trust is very effective, which is explained in this study as one of the main components. On the other hand, studies have shown that in most countries, maintaining and promoting public trust is very important and necessary, and in this direction, in addition to clear and timely information, there should be a two-way interaction and attention should be paid to understanding the risk and people’s needs (Boholm 2019).

Active information production as well as completing the information gap from before the disaster to the rehabilitation phase, in addition to increasing public trust, will lead to the management of social networks and rumours, which will help manage the stress and turmoil caused by crises (Sezgin, Karaaslan & Ersoy 2020). Content producing, there must be an intelligent data collection system to fill the information gap at the intra-organisational and community levels (Bernard et al. 2021; Johnson & Chess 2006) in many communities educating people to correct rumours, manage public opinion and reduce the burden. Emotional and psychological events and disasters are part of precrisis risk communication programmes (Granatt 2004; Zhang et al. 2021).

Finally, from the perspective of the researchers in this study, assessing the relationship between disaster risk understanding, the components of the communication process and describing the relationship between these elements, including how the process evolves and how structures relate to each other and outcomes, should be clear.

In this study, monitoring focuses on evaluation, accreditation and documentation. Monitoring and evaluation are one of the main elements of management and are of great importance in risk communication also. However, Dickmann et al. (2016), points out that communication interventions in public health emergencies (such as infectious disease outbreaks) are increasingly recognised as a critical determinant of success in preparedness, response, and recovery. It is possible that its impact can be measured through the evaluation of related performance parameters (Hooker & Leask 2020). In this framework and similar studies, only information listening, the communication framework including actions, key messages and strategies, and finally coordination at different levels are evaluated (Dickmann et al. 2016).

According to this research, the establishment of communication processes, preparation and provision of communication infrastructure from simple to advanced equipment, such as the use of various structured formats for communication, artificial intelligence for communication and determining online and offline communication support systems, can achieve goals. Internal and interdepartmental coordination and organisation and increasing community engagement increases the organisational preparedness to deal with disasters.

Another dimension of the risk communication process is to establish an effective, appropriate and continuous relationship with the community, which requires the establishment of communication between them, the formation of community networks and the use of key persons at the community level (Hobbs et al. 2004; Savoia et al. 2017; Venkatraman & Manoharan 2021). Tambo et al. (2021) showed in their study that social and global solidarity requires strong governance, broad community participation and sufficient trust to enhance preparedness and early response to epidemics (Tambo et al. 2021), which emphasises the findings of the current study. Gathering information and providing infrastructure based on artificial intelligence and machine learning algorithms will be useful and effective in promoting risk communication interventions and building public trust (Ghomian et al. 2018; Seeger et al. 2018; Zhang et al. 2021). The recording, storage and dissemination of information through the appropriate platform will help to promote adaptive prevention and control programmes for emerging epidemics, evidence-based decision-making, integration of data and models for information and the provision of effective and sustainable risk communication strategies (Johnson & Chess 2006).

## Conclusion

In general, disaster risk communication has many and varied components because of its vital and effective functions, which are fully enumerated in this study. One of the reasons for the failure of communication programmes is the risk of interdisciplinary approaches in this field, which has caused some important elements to be ignored. The development of programmes, protocols, guidelines and instructions can use all the elements extracted in this study, in addition to strengthening risk communication, preventing the entry of managerial and policy tastes as much as possible and leading to internal and external coordination. Increasing coordination in all steps of risk communication will increase social participation and public trust as the first step in changing the positive behaviour of society in the field of health. This study has extracted the risk communication components with an all-hazard approach, but it is suggested to conduct a specific investigation regarding the major disaster.

Implementation of interactive risk communication and information on the disaster risk reduction phase based on the community needs can develop knowledge and lead to behaviour changes. The ecology of risk communication should be given the attention of policymakers in this field. Mass information production will not have much effect regardless of the knowledge, experience, values, economic, social and religious status of the society. Laws and ethical principles related to disaster risk communication should be formulated, keeping in mind the spread of social networks and the increase of infodemics.

### Strengths and limitations

One of the strengths of this research was the study of risk communication elements in the time of coronavirus disease 2019 (COVID-19), which itself is a result of the existing conditions and the use of different policies, leading to the explanation of all the components without any shortcomings. Mind map software was also used to provide a big picture to familiarise the audience with all aspects of disaster risk communication. One of the limitations of this research was the lack of access to the full text of some articles and books, the breadth of the content and the number of articles included in the study.
